# Substrate-dependent modulation of the leukotriene A_4_ hydrolase aminopeptidase activity and effect in a murine model of acute lung inflammation

**DOI:** 10.1038/s41598-022-13238-6

**Published:** 2022-06-08

**Authors:** Kyung Hyeon Lee, Nadia Fazal Ali, Soo Hyeon Lee, Zhimin Zhang, Marie Burdick, Zachary J. Beaulac, Greg Petruncio, Linxia Li, Jiangdong Xiang, Ezra M. Chung, Kenneth W. Foreman, Schroeder M. Noble, Yun M. Shim, Mikell Paige

**Affiliations:** 1 Department of Chemistry & Biochemistry, George Mason University, 10920 George Mason Circle, Manassas, VA 20110 USA; 2 Wound Infections Department, Bacterial Diseases Branch, Walter Reed Army Institute of Research, 503 Robert Grant Ave, Silver Spring, MD 20910 USA; 3 Division of Pulmonary and Critical Care Medicine, Department of Medicine, University of Virginia, PO Box 800546, Charlottesville, VA 22908 USA; 4 Department of Obstetrics and Gynecology, Seventh People’s Hospital of Shanghai University of Traditional Chinese Medicine, 358 Datong Road, Shanghai, 200137 China; 5 Department of Obstetrics and Gynecology, Shanghai General Hospital, Shanghai Jiao Tong University School of Medicine, Shanghai, 200080 China; 6 STCube Pharmaceutical, Inc., 401 Professional Dr, Gaithersburg, MD 20879 USA

**Keywords:** Enzyme mechanisms, Mechanism of action, Small molecules, Enzyme mechanisms, Structural biology

## Abstract

The aminopeptidase activity (AP) of the leukotriene A_4_ hydrolase (LTA_4_H) enzyme has emerged as a therapeutic target to modulate host immunity. Initial reports focused on the benefits of augmenting the LTA_4_H AP activity and clearing its putative pro-inflammatory substrate Pro-Gly-Pro (PGP). However, recent reports have introduced substantial complexity disconnecting the LTA_4_H modulator 4-methoxydiphenylmethane (4MDM) from PGP as follows: (1) 4MDM inhibits PGP hydrolysis and subsequently inhibition of LTA_4_H AP activity, and (2) 4MDM activates the same enzyme target in the presence of alternative substrates. Differential modulation of LTA_4_H by 4MDM was probed in a murine model of acute lung inflammation, which showed that 4MDM modulates the host neutrophilic response independent of clearing PGP. X-ray crystallography showed that 4MDM and PGP bind at the zinc binding pocket and no allosteric binding was observed. We then determined that 4MDM modulation is not dependent on the allosteric binding of the ligand, but on the N-terminal side chain of the peptide. In conclusion, our study revealed that a peptidase therapeutic target can interact with its substrate and ligand in complex biochemical mechanisms. This raises an important consideration when ligands are designed to explain some of the unpredictable outcomes observed in therapeutic discovery targeting LTA_4_H.

## Introduction

Leukotrienes regulate complex inflammatory responses by interacting with the host and external stimuli as paracrine mediators^1–3^. Induction of these pathways involves the processing of arachidonic acid to biologically active leukotrienes^1,4^. Leukotriene A_4_ hydrolase (LTA_4_H) is a zinc metalloenzyme that stands out with unique dual functionalities that include peptidase activity in addition to lipid metabolism. As an epoxide hydrolase (EH), LTA_4_H converts leukotriene A_4_ (LTA_4_) to leukotriene B_4_ (LTB_4_). Increased production of LTB_4_ induces neutrophilic inflammation found in chronic obstructive pulmonary disease (COPD), sepsis, and acute lung injury^5–8^. As an aminopeptidase (AP), LTA_4_H hydrolyzes natural ligands such as dynorphin, enkephalin, and the tripeptide proline-glycine-proline (PGP), of which PGP has garnered significant interest^1,9–15^. PGP is an extracellular matrix-driven neutrophil chemoattractant, which is generated from collagen through a multistep proteolytic pathway involving matrix metalloproteases 8 and 9 (MMP-8 and MMP-9) and prolyl endopeptidase (PE)^7,38,52^. The current understanding is that LTA_4_H AP activity resolves neutrophilic inflammation specifically by digesting and clearing PGP since accumulation of PGP activates the CXCR2 receptors on neutrophils^5,16–18^. Therefore, bi-functional EH and AP activities undergird the intricate pro- and anti-inflammatory regulation, respectively, exerted by LTA_4_H.

Snelgrove reported that lipopolysaccharide (LPS) exposure causes bioproduction of PGP and acute lung injury^5^. Unlike cigarette smoke exposure, LPS exposure does not suppress endogenous LTA_4_H AP activity, and therefore, active LTA_4_H AP clears PGP after exposure to LPS in murine lung^5^. Numao reports additional observations, which show that PGP does not induce inflammatory responses at micromolar concentrations found in the murine air-pouch model of inflammation^19^. This report on PGP is puzzling since several independently corroborating reports demonstrate that the in vivo levels of PGP correlate with host inflammatory responses and outcomes^5,6,20^. These contradicting reports also suggest that cigarette smoke causes lung injury by an accumulation of acrolein and acidification of the airways, which result in an accumulation of PGP in the lungs via suppression of LTA_4_H AP activity^6,8,21^. In murine models of lung injury, reducing PGP levels in the lung by 4-methoxydiphenylmethane (4MDM) correlates with the resolution of neutrophilic inflammation and other beneficial therapeutic effects^6,20^. 4MDM selectively augments the LTA_4_H AP activity and does not affect the EH activity^6,20^. 4MDM has minimal off-targeting effects reported by our group as demonstrated in an LTA_4_H knockout murine model^6^. We have demonstrated that treatment with 4MDM restores the LTA_4_H AP activity and prevents PGP accumulation by cigarette smoke exposure^6^. To clarify the contradicting reports on PGP, we set forth to conduct studies using 4MDM as a pharmaceutical tool to interrogate the biochemistry of the LTA_4_H AP activity.

First, we characterized the effects of selectively augmenting LTA_4_H AP activity on pulmonary inflammation induced by LPS. We have taken advantage of this unique aspect with the LPS murine model to determine the effect of selectively augmenting LTA_4_H AP activity with 4MDM in a PGP-independent model for pulmonary inflammation with 4MDM. Since PGP interaction with LTA_4_H is not completely characterized biochemically, our study is mainly focused on the AP activity of LTA_4_H in the presence of 4MDM. In addition to PGP, dynorphin and enkephalin are peptide-based substrates for the LTA_4_H AP activity^9–12^. As reported by Orning and co-workers, LTA_4_H hydrolyzes tripeptides containing an N-terminal arginine more efficiently than other peptide substrates such as dipeptides, tetrapeptides, pentapeptides, and tripeptides with non-arginine N-termini^22^. Therefore, LTA_4_H can be considered an arginine N-aminotripeptidase^22^. In order to gain more insight into the substrate-specific properties of LTA_4_H AP activity, we determined the effect of 4MDM on the kinetic mechanisms for hydrolysis of PGP, Ala-*p*NA, Arg-*p*NA, and Pro-*p*NA. Lastly, we reported the first X-ray crystal structures of LTA_4_H in complex with 4MDM and LTA_4_H bound to 4MDM and N-(4-oxo-4-pyrrolidinyl-butanoyl)-proline (OPB-Pro), a non-hydrolyzable analogue of PGP.

## Results

### Regulation of LTA_4_H bifunctionality in the murine model of acute lung inflammation and injury induced by intra-nasal LPS

**Figure 1:**
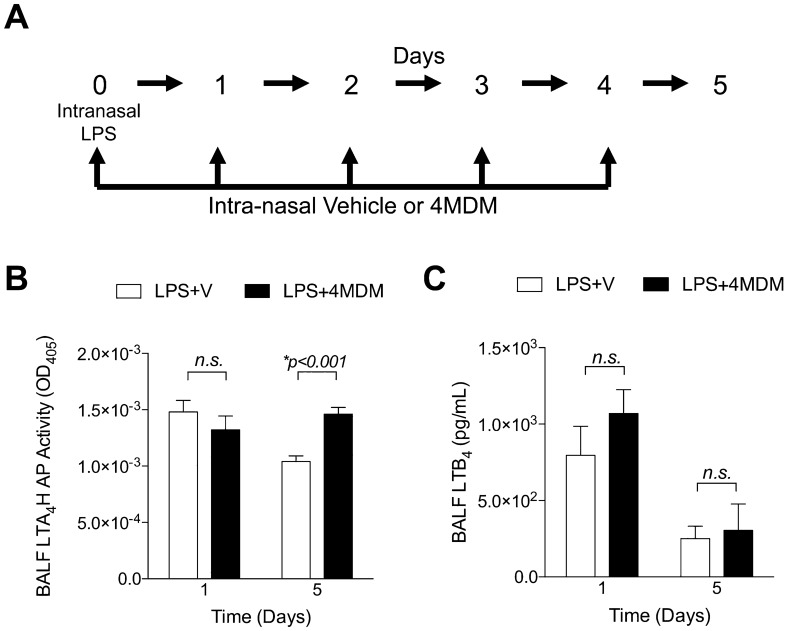
(**A**) Experimental design of the murine model of acute lung injury induced by intranasal lipopolysaccharide (LPS). (**B**) LTA_4_H aminopeptidase activity in the bronchoalveolar lavage fluid. (**C**) LTA_4_H epoxide hydrolase activity in the bronchoalveolar lavage fluid reflected by the levels of LTB_4_. Veh = cyclodextrin vehicle in phosphate-buffered saline. 4MDM = drug in the vehicle. *Significant *p*-values by Holm-Sidak comparison. n.s., not significant.

The experimental design of the murine model is shown in Fig. 1A. Mice were treated daily from Days 0 to 4 with intranasal (IN) 4MDM after being exposed to IN LPS on Day 0. LTA_4_H AP activity was significantly elevated after five days of 4MDM treatment over that of the vehicle (Fig. 1B). Levels of LTB_4_ in the bronchoalveolar lavage fluid (BALF) were comparable between the 4MDM- and vehicle-treated animals (Fig. 1C). These studies indicated that 4MDM treatment selectively enhanced the LTA_4_H AP activity without affecting the EH activity in the murine model of LPS-induced acute lung injury. PGP concentration was assessed in the whole lung BALF collected from two treatment cohorts after LPS exposure (vehicle vs. 4MDM) on Days 1 and 5. While the PGP levels in the BALF were above the level of detection, they were well below the lower limit of quantification except in the BALF samples from the cohort treated with 4MDM for five days. In this model, the concentration of PGP was at a level where its biological contribution was believed to be trivial (Tables S1 and S2).

**Figure 2:**
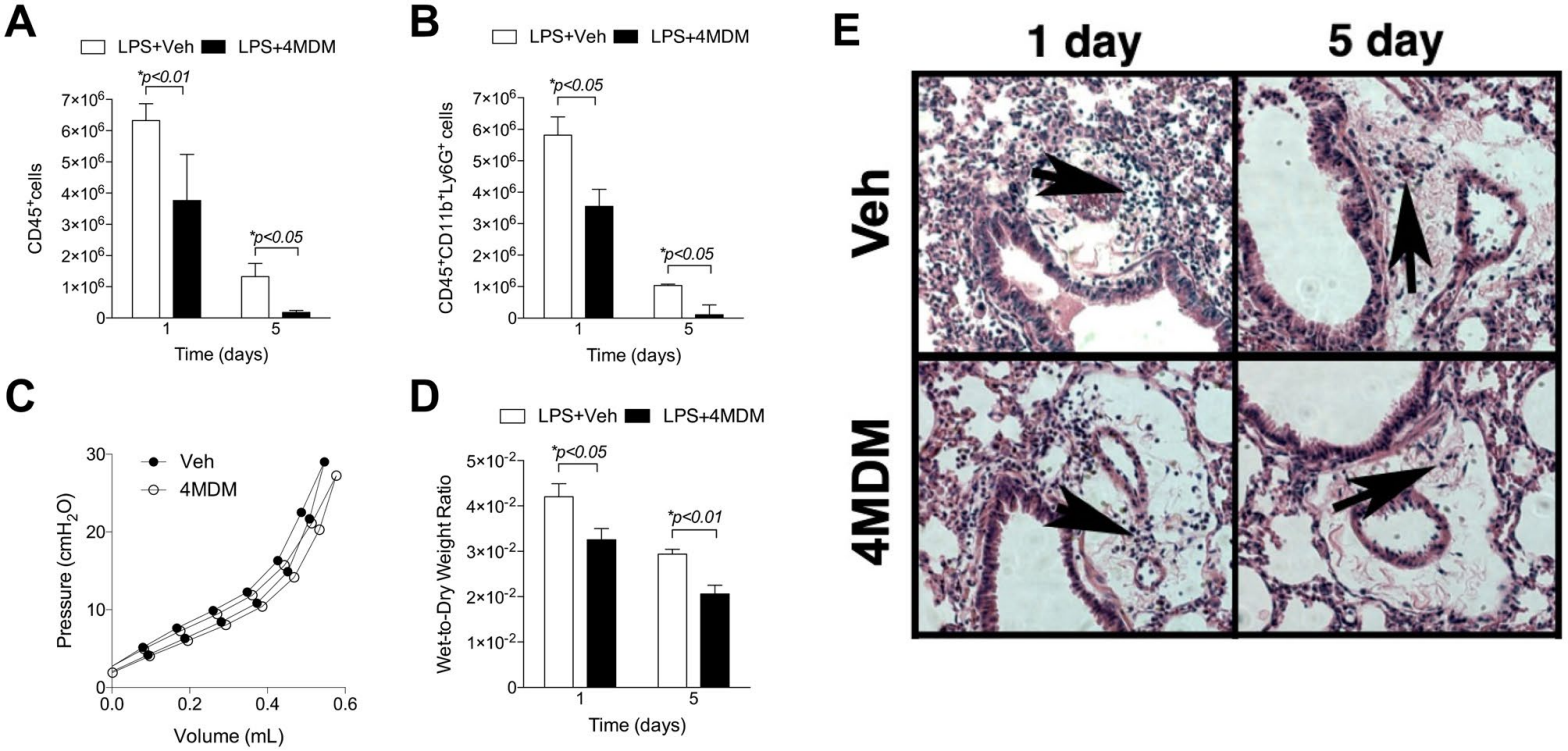
(**A**) CD45^+^ leukocytes in the bronchoalveolar lavage fluid by flow cytometry. (**B**) CD45^+^CD11b^+^Ly6G^+^ leukocytes in the bronchoalveolar lavage fluid by flow cytometry. (**C**) Premortem Pressure–Volume loop measured by Sireq Flexivent on Day 5. (**D**) Lung wet-to-dry weights ratio on Days 1 and 5. (**E**) H&E staining of the bronchovesicular bundle with vehicle or 4MDM treatment. Arrow indicates leukocytes infiltrating the peri-bronchial vascular bundles. Veh = cyclodextrin vehicle in phosphate-buffered saline. 4MDM = drug in the vehicle. *Significant *p*-values by Holm-Sidak comparison. n.s., not significant.

4MDM treatment consistently reduced the number of CD45^+^ leukocytes and CD45^+^CD11b^+^Ly6G^+^ neutrophils in the LPS-exposed lungs on Days 1 and 5 (Fig. 2A,B), while LTA_4_H activity in BALF was significantly augmented on Day 5 (Fig. 1B). LTA_4_H activity measured in the BALF may not fully reflect that of activity in lung tissue. The severity of acute lung injury was assessed by wet-to-dry lung weight ratio and Sireq Flexivent pressure–volume loop. 4MDM treatment maintained the lung to be more compliant by the premortem pressure–volume loop, consistent with less water content and less severe acute lung injury (Fig. 2C,D). Representative H&E staining shows significantly fewer leukocytes infiltrating the peri-bronchial areas (arrow) with 4MDM treatment than with vehicle alone (Fig. 2E).

### Enzyme kinetics of LTA_4_H for the hydrolysis of PGP, Arg-pNA, Ala-pNA, and Pro-pNA in the presence of 4MDM

**Figure 3:**
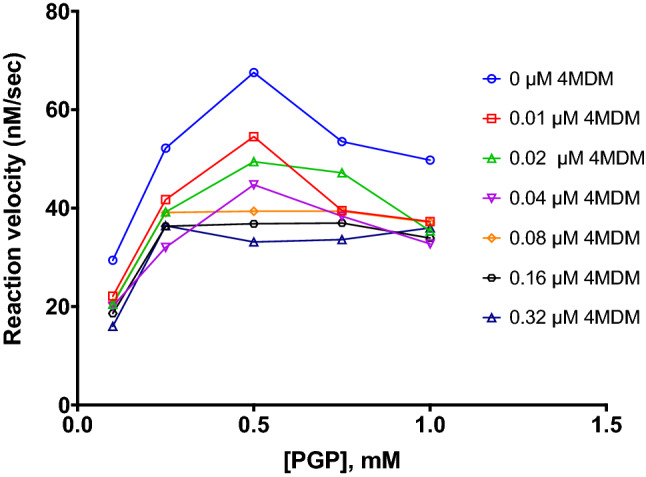
Reaction velocity plot of LTA_4_H-mediated hydrolysis of PGP. Reaction velocity plot showing the modulation of PGP-induced (substrate-induced) inhibition in the presence of 4MDM. Each sample was prepared in twenty replicates (n = 20), and the coefficient of variation is within 5%. Data shown in the figure is the average of twenty replicates.

The reaction velocity plots of PGP hydrolysis were determined at escalating concentrations of 4MDM (Fig. 3). The data is consistent with substrate-induced inhibition at increasing substrate concentrations. Our data suggested that a substrate-enzyme–substrate (SES) complex formed, which would involve the addition of a second PGP molecule to the LTA_4_H-PGP complex^23,24^. 4MDM accentuated PGP-induced inhibition in a dose-dependent manner.

**Figure 4:**
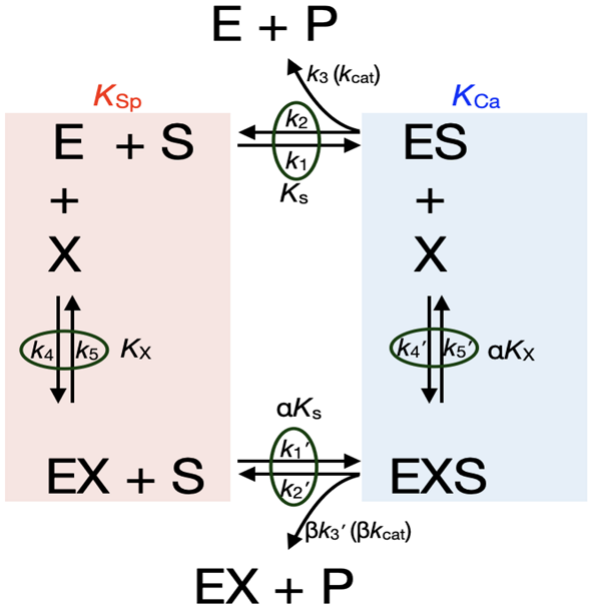
The kinetic scheme of non-essential mixed type activation and rate constants (Baici, 2015). The *k*_1_, *k*_2_, *k*_3_, *k*_4_, and *k*_5_ are rate constants, and the unit with *k*_2_, *k*_3_, and *k*_5_ in s^−1^, and the unit of *k*_1_ and *k*_4_ in M^−1^ s^−1^. The corresponding dissociation constants *K*_S_ and *K*_X_, in M, equal *k*_2_/*k*_1_ and *k*_5_/*k*_4_, respectively. The Michaelis–Menten constant *K*_m_ equals (*k*_2_ + *k*_3_)/*k*_1_ with units of M. The catalytic constant *k*_cat_ (*k*_3_) is in s^−1^. α and β are dimensionless positive coefficients. *K*_Sp_ and *K*_Ca_ refer to the specific (competitive) and catalytic (uncompetitive) modifications, respectively. The catalytic efficiency *k*_cat_/*K*_m_ of the enzyme is used for comparing the relative rates of enzyme activity acting on the substrate with or without modifiers.

**Figure 5:**
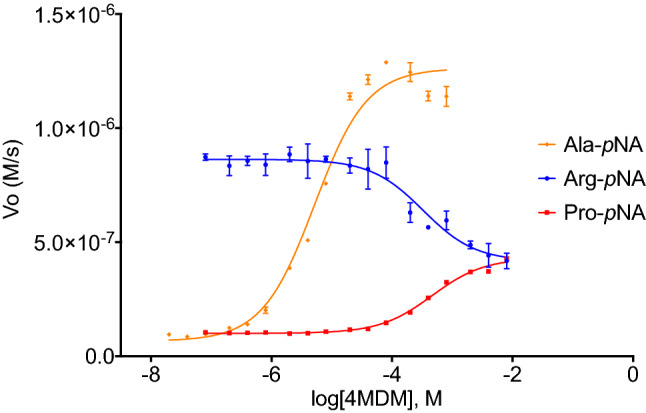
The AC_50_ and IC_50_ curves for 4MDM were determined using four different substrates Ala-*p*NA (orange), Arg-*p*NA (blue), and Pro-*p*NA (red). Hydrolysis of Arg-*p*NA by LTA4H was inhibited in the presence of 4MDM with an IC_50_ of 328.10 μM. Hydrolysis of Ala-*p*NA and Pro-*p*NA was activated by 4MDM with an AC_50_ of 4.83 μM and 462.81 μM, respectively. All assays were run under the same buffer conditions with 4MDM concentrations between 0 and 8000 μM. The bars represent ± standard deviation (n = 6).

**Figure 6:**
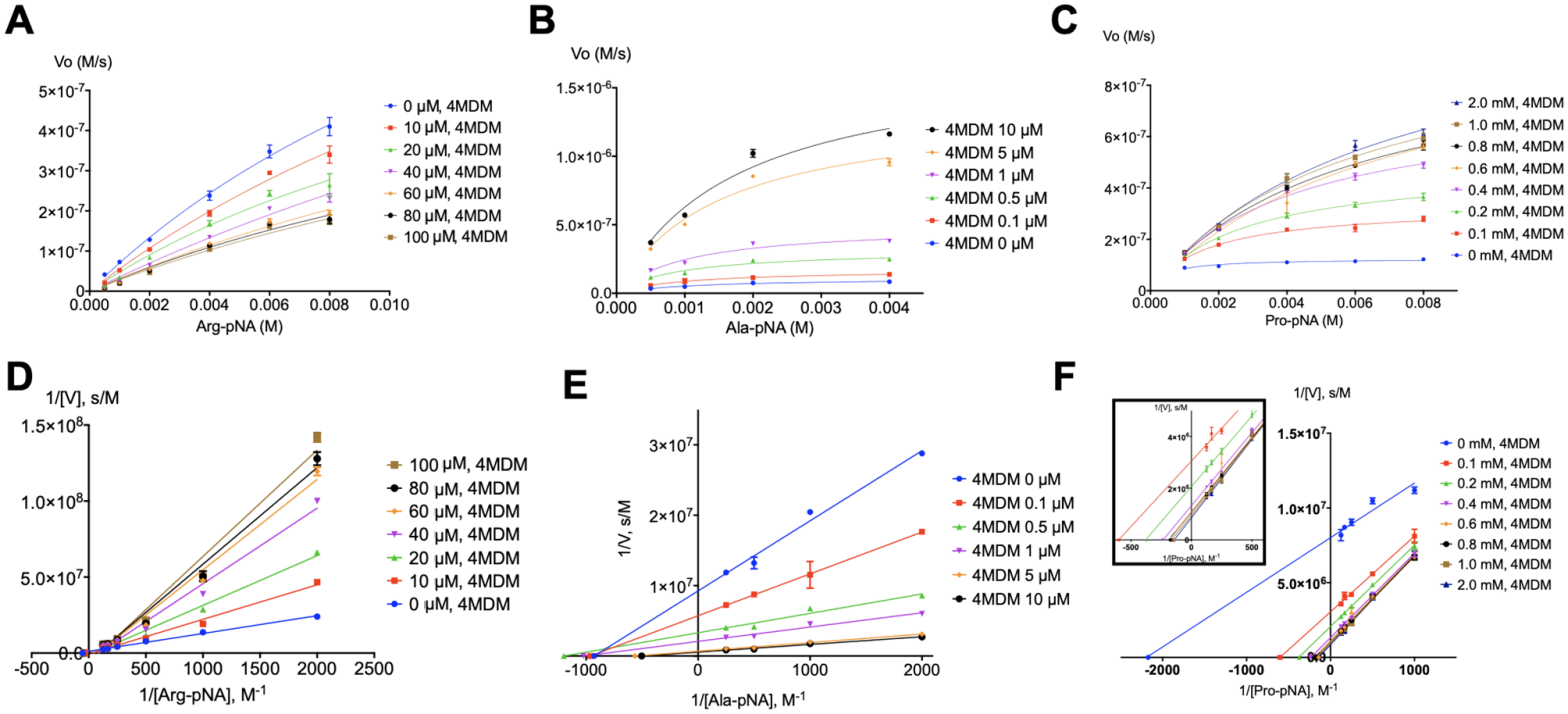
Enzyme kinetic data represented in the Michaelis–Menten plot (**A**, **B**, **C**) and Lineweaver–Burk plot (**D**, **E**, **F**). In the presence of 4MDM, the hyperbolic mixed predominantly specific inhibition is shown with Arg-*p*NA (**A**, **D**), the hyperbolic mixed predominantly catalytic activation with Ala-*p*NA (**B**, **E**), and the hyperbolic catalytic activation with Pro-*p*NA (**C**, **F**). The bars represent ± standard deviation from six replicates (n = 6).

Enzyme kinetics studies were performed to elucidate the kinetic mechanism of LTA_4_H-mediated hydrolysis of Arg-*p*NA, Ala-*p*NA, and Pro-*p*NA at escalating concentrations of 4MDM (Figs. 4, 5, 6). 4MDM induced hyperbolic predominantly specific inhibition in the presence of Arg-*p*NA, hyperbolic mixed predominantly catalytic activation with Ala-*p*NA, and hyperbolic catalytic activation with Pro-*p*NA.

These studies revealed the effect of 4MDM on LTA_4_H kinetics by perturbing the equilibrium coupling constant α and the enzyme kinetic catalytic constant β. Decreasing values for α signify increasing stabilization of the enzyme-modifier-substrate (EXS) complex where X is 4MDM, E is LTA_4_H, and S is amino acid-*p*NA substrate (Fig. 4). Therefore, 4MDM is likely an unsuitable activator for potential R-X-X substrates, because it inhibits Arg-*p*NA binding competitively by stabilizing the formation of the EX complex. Given the high α value for hydrolysis of Arg-*p*NA in the presence of 4MDM, 4MDM and Arg-*p*NA cannot simultaneously bind to LTA_4_H, presumably due to the large size of the Arg side chain. However, 4MDM predominantly activates Ala-*p*NA hydrolysis by increasing catalytic turnover (β > 1) for the EXS complex^25^. In contrast to Arg- and Ala-*p*NA mechanisms, 4MDM "uncompetitively" activates Pro-*p*NA hydrolysis where the equilibrium coupling constant α equals the kinetic catalytic constant β (α = β). The catalytic specificities and kinetic parameters for hyperbolic enzyme modifications of Arg-, Ala-, Pro-*p*NA are shown in Tables 1 and 2^23^. These studies suggested that for activation, 4MDM does not universally activate the LTA_4_H enzyme and its ability to activate or inhibit hydrolysis highly depends on the nature of the substrate. Notably, 4MDM affects the ES complex by contributing to partial predominantly catalytic activation (Ala-*p*NA) or exclusive catalytic activation (Pro-*p*NA).

**Table 1:** Catalytic efficiency (*k*_*cat*_/K_M_) of 4MDM with Ala-*p*NA, Arg-*p*NA, and Pro-*p*NA.

Arg-*p*NA				Ala-*p*NA				Pro-*p*NA			
4MDM [µM]	*k*_*cat*_ (s^−1^)	K_M_ (mM)	*k*_*cat*_/K_M_ (mM^−1^ s^−1^)	4MDM [µM]	*k*_*cat*_ (s^−1^)	K_M_ (mM)	*k*_*cat*_/K_M_ (mM^−1^ s^−1^)	4MDM [µM]	*k*_*cat*_ (s^−1^)	K_M_ (mM)	*k*_*cat*_/K_M_ (mM^−1^ s^−1^)
0	24.35 ± 2.67	18.79 ± 2.73	1.30	0	1.74 ± 0.07	1.60 ± 0.12	1.08	0	0.19 ± 0.01	0.84 ± 0.05	0.23
10.0	24.62 ± 3.99	24.26 ± 4.95	1.02	0.1	3.01 ± 0.13	0.98 ± 0.12	3.07	100.0	0.65 ± 0.03	1.89 ± 0.25	0.34
20.0	15.59 ± 3.01	17.70 ± 4.58	0.88	0.5	5.54 ± 0.23	0.91 ± 0.11	6.06	200.0	0.93 ± 0.08	2.90 ± 0.62	0.32
40.0	24.42 ± 7.31	37.89 ± 13.19	0.64	1.0	8.68 ± 0.36	1.03 ± 0.12	8.46	400.0	1.25 ± 0.06	3.92 ± 0.40	0.32
60.0	16.22 ± 3.99	28.31 ± 8.50	0.57	5.0	23.91 ± 1.04	1.52 ± 0.16	15.71	600.0	1.36 ± 0.09	4.32 ± 0.63	0.32
80.0	12.45 ± 3.07	21.98 ± 6.96	0.57	10.0	30.27 ± 1.50	1.75 ± 0.19	17.26	800.0	1.53 ± 0.07	4.91 ± 0.46	0.32
100.0	13.76 ± 3.90	26.66 ± 9.33	0.52					1000.0	1.56 ± 0.24	4.81 ± 1.55	0.32
								2000.0	1.87 ± 0.15	5.49 ± 0.84	0.34

*k*_*cat*_ and K_M_ values were analyzed using GraphPad Prism 7.0. Data are represented as mean ± standard deviation from six replicates and the coefficient of variation is within 5%.

**Table 2:** Calculated kinetic parameters of each substrate mechanism by LTA_4_H AP activity.

	Arg-*p*NA	Ala-*p*NA	Pro-*p*NA
α	30.39	0.82	7.88
β	0.09	25.4	7.88
*k*_cat_ (s^−1^)	24.35 ± 2.67	1.74 ± 0.15	0.19 ± 0.01
Mechanism	Hyperbolic predominantly specific inhibition	Hyperbolic predominantly catalytic activation	Hyperbolic catalytic activation

### Crystal structure of LTA_4_H in complex with 4MDM and 4MDM:OPB-Pro

**Figure 7:**
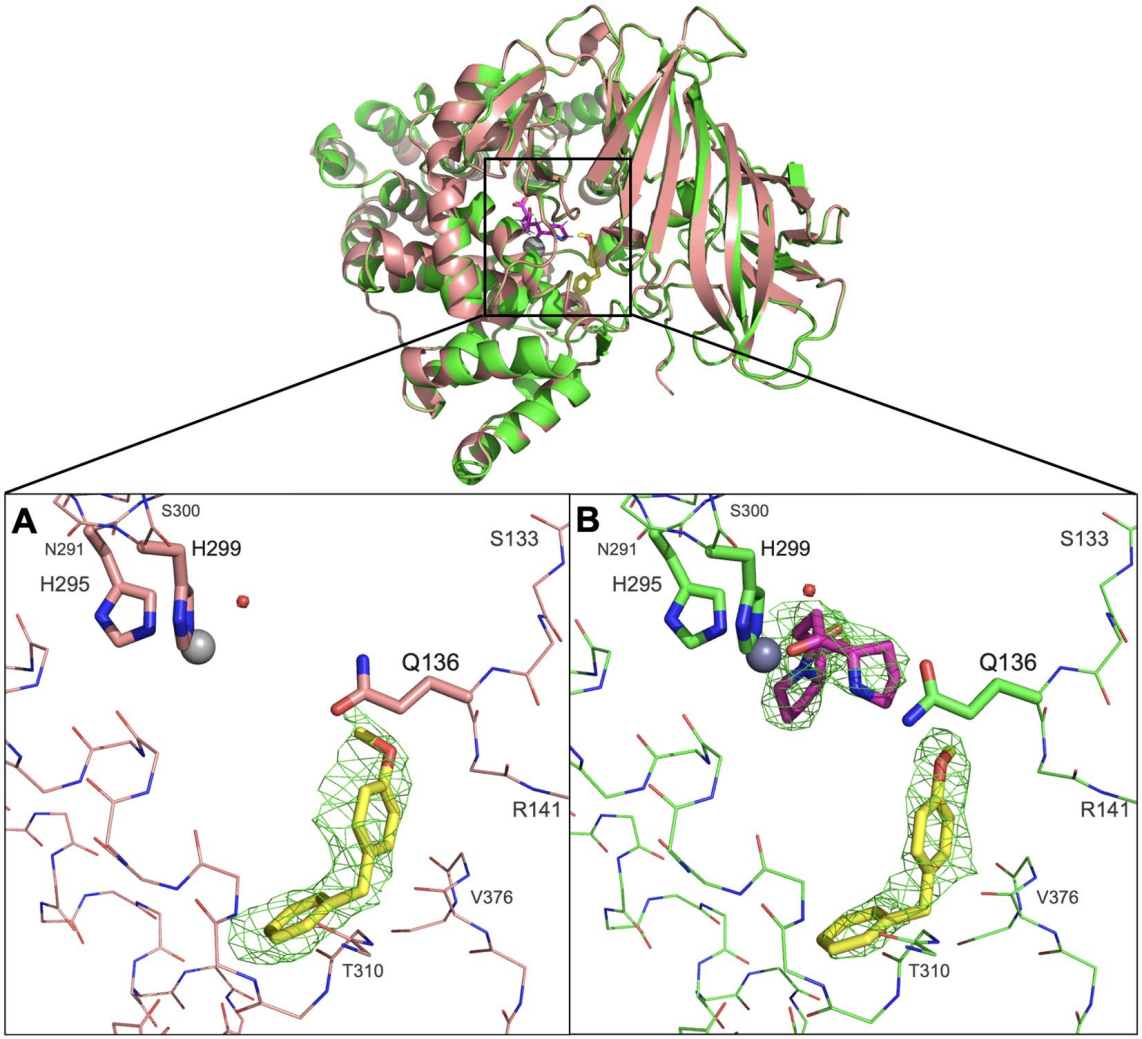
Superimposed crystal structures of LTA_4_H:4MDM and LTA_4_H:4MDM:OPB-Pro complexes (top). (**A**) A close-up view of the LTA_4_H binding pocket with 4MDM (yellow) bound within the short end of the L-shaped hydrophobic pocket of LTA_4_H (salmon) is shown. (**B**) A close-up view of the LTA_4_H binding pocket with both 4MDM (yellow) and OPB-Pro bound (green) is shown. Active site Zn^2+^ and coordinating His residues are indicated. The direction of the Gln-136 side chain points toward the hydrophilic binding site with respect to water. The direction of the methoxy group in 4MDM is rotated in the tri-complex. Background residues were deleted to enhance the visibility of the binding site except for residues from Ser-133 to Arg-141, from Asn-291 to Ser-300, and from Thr-310 to Val-376 are shown in line rendering.

Human recombinant LTA_4_H was co-crystallized with 4MDM (Fig. 7A) and 4MDM:OPB-Pro (Fig. 7B), where OPB-Pro is a non-hydrolyzable analog of PGP. The full-length structure of LTA_4_H in complex with 4MDM was refined at 2.9 Å to R-work of 18% and R-free of 21%. The LTA_4_H-4MDM complex crystallized in space group P3_2_, with three molecules in the asymmetric unit. As shown in the M1 metallopeptidase HEXXH-(X)_18_E motif, the Zn^2+^ ion was coordinated with H295, E296 H299, and the carboxylic acid side chain oxygen atom of E318^26^. The 2.8 Å X-ray crystal structure of LTA_4_H in complex with 4MDM and OPB-Pro was refined to R-work of 19.9% and R-free of 22.9%. OPB-Pro and 4MDM were both bound in each of the three molecules of LTA_4_H in the asymmetric unit. The structure revealed that the OPB-Pro and 4MDM bound within the LTA_4_H active site, but at a significant distance of > 4.0 Å away from each other. The OPB-Pro was bound in the AP active site with its prolyl carbonyl group interacting with the zinc atom. 4MDM bound to the hydrophobic pocket of LTA_4_H in a similar orientation as the LTA_4_H:4MDM complex structure, and was stabilized by van der Waals interactions.

The methoxy group of 4MDM in the LTA_4_H:4MDM complex was oriented toward the Zn^2+^ cation, but is oriented toward Q136 in the LTA_4_H:4MDM:OPB-Pro complex. At the same time, the oxygen atoms of the methoxy group of 4MDM in the LTA_4_H:4MDM complex is shifted by 1.3 Å in the LTA_4_H:4MDM:OPB-Pro complex. 4MDM in the LTA_4_H:4MDM:OPB-Pro complex bound slightly deeper into the hydrophobic binding pocket, and shifted by 0.5 Å toward F362. The position of the side chain of Q136 marked a notable difference between the LTA_4_H:4MDM complex and the published LTA_4_H:OPB-Pro structure (PDB ID: 4MS6)^27^. The polar side chain of Q136 was oriented toward the aminopeptidase active site in the LTA_4_H:4MDM complex, whereas it was oriented toward the hydrophobic cavity in the LTA_4_H:OPB-Pro structure (PDB ID: 4MS6)^27^. Hydrogen bonding interactions were present between the O^ε1^ atom of Q136 and the Cγ atom of OPB-Pro. In the published LTA_4_H:ARM1:OPB-Pro structure (PDB ID: 4MKT)^27^, two rotamers were observed for the side chain of Q136, while the side chain of Q136 in the LTA_4_H:4MDM:OPB-Pro structure showed only one orientation, where the side chain orients toward the catalytic Zn^2+^ cation. Also, the crystal structure of LTA_4_H:4MDM showed that Q136 was oriented towards the Zn^2+^ cation, which has the same orientation shown in the structure of LTA_4_H:4-OMe-ARM1 complex (PDB ID: 6O5H)^25^. This additional structural information provided by the LTA_4_H:4MDM and LTA_4_H:4MDM:OPB-Pro complexes indicates that Q136 could have a role in tripeptide substrate recognition and catalytic turnover by maintaining rotational freedom^28^. In both 4MDM bound structures, the catalytic water molecule was presented near the Zn^2+^ ion and interacted with E296. Our interpretation is that the Q136 side chain maintains the catalytic water molecule interaction with E296 while simultaneously engaging in multiple hydrophobic interactions via its rotational freedom. These aspects are critical determinants of peptidase potentiation by 4MDM. Also, two non-catalytic water molecules form hydrogen bond interactions with Q136, N317 and E318 in the LTA_4_H:ARM1:OPB-Pro (PDB ID: 4MKT)^27^, LTA_4_H:OPB-Pro (PDB ID: 4MS6)^27^, LTA_4_H:ARM1 (PDB ID: 4L2L)^27^ and LTA_4_H:4MDM complexes but are absent in the LTA_4_H:4MDM:OPB-Pro complex (Fig. S3). We analyzed that the LTA_4_H:4MDM:OPB-Pro complex created a lower dielectric environment, which induced the release of these non-catalytic water molecules. The loss of water molecules could afford more favorable binding entropies for nonpolar ligand binding, provided steric clashes are avoided.

## Discussion

Numerous human pathologies correlate with dysfunctional LTA_4_H activities that result in an accumulation of LTB_4_ and PGP^5,8,14^. Research studies suggest that PGP is a pro-inflammatory matrikine that is produced during host tissue injury^18^. While host proinflammatory responses seem to be strongly associated with elevated levels of PGP, a recent study questions the biological activity of PGP. Numao and co-workers suggest that the anti-inflammatory LTA_4_H AP activity may result from clearance of substrates other than PGP, implying that even at high levels, PGP might be biologically less relevant to inflammatory signaling^19^. Therefore, we employed a murine model of LPS-induced pulmonary neutrophilic inflammation to ascertain the biology of LTA_4_H AP activity independent of PGP. Previous studies have shown that IN LPS induces acute neutrophilic inflammation in lungs while simultaneously inducing the LTA_4_H enzyme levels^29,30^. Past studies showed that PGP levels declined rapidly within 24 h due to endogenous LTA_4_H AP activity after exposure to LPS^5^. Numao also reported that the levels of PGP observed in the murine lung exposed to LPS did not cause significant neutrophilic inflammation^19^. Therefore, we concluded that the murine pulmonary inflammation model induced by LPS was a suitable model to ascertain if at least a part of the LTA_4_H AP biology is unrelated to PGP. In this model, perturbation of the LTA_4_H AP activity by 4MDM will provide additional corroboration. We construed that if the LTA_4_H AP-mediated hydrolysis of PGP is a significant anti-inflammatory pathway, 4MDM should not alter LPS-induced neutrophilic inflammation because PGP, the putative target of the LTA_4_H AP activity, is cleared rapidly after LPS exposure. Our study demonstrated that LTA_4_H AP activity was selectively augmented by 4MDM when Ala-*p*NA or Pro-*p*NA were used as reporters, which corresponded to significantly reduced airway neutrophilia caused by IN LPS throughout the 5-day observation period. PGP levels in BALF were low or undetectable, suggesting that the biological effects of the augmented LTA_4_H AP activity is independent of PGP. Beneficial effects were also independent of LTB_4_ levels. These results led us to conclude that augmentation of the LTA_4_H AP activity brings broader biological effects beyond the clearance of PGP. These observations bring forth a speculation that the LTA_4_H AP activity is not exclusive to the clearance of PGP, and therefore raises the need for detailed enzymatic studies on the LTA_4_H AP activity with substrates other than PGP.

Findings from our studies reveal several previously underappreciated biochemical mechanisms. First, PGP interacts with LTA_4_H AP by a substrate-induced inhibition mechanism. Under this mechanism, increasing concentrations of PGP would negatively feedback the LTA_4_H AP activity. Besides accelerating PGP accumulation, the substrate-induced inhibition may cause persistent inflammation by inhibiting LTA_4_H AP activity, assuming robust LTA_4_H AP activity is required to maintain the anti-inflammatory functions of LTA_4_H. Second, the enzyme kinetics significantly diverged depending on substrates. Arg-*p*NA, Ala-*p*NA, and Pro-*p*NA interact with the LTA_4_H AP substrate site under three distinctive enzymatic mechanisms in the presence of 4MDM. While 4MDM primarily inhibited hydrolysis of Arg-*p*NA, 4MDM enhanced the hydrolysis of Ala-*p*NA and Pro-*p*NA. Of these three substrates, the enzyme kinetics of Pro-*p*NA was most intriguing as it followed a rarely observed hyperbolic catalytic activation (HCaA) mechanism, which is analogous to an “uncompetitive inhibition” mechanism where the modulator has higher affinity for the enzyme–substrate complex than to the enzyme. Surprisingly, PGP was further inhibited by the presence of 4MDM, which showed us that the structure of the N-terminus of the reporter group is insufficient to report the enzyme kinetics for corresponding peptides. Regardless, we speculate that the effect of PGP hydrolysis may not be relevant for our preclinical model.

Comparisons with previously published X-ray crystal structures of LTA_4_H:R-X-X tripeptides complexes and the LTA_4_H:4MDM complex can help explain our enzyme kinetics data from a structural perspective. X-ray crystal structures of LTA_4_H complexes demonstrate that RSR and RAR tripeptides bind the GXMEN motif in an extended β strand conformation^31^. The structures also reveal that Asp375, a key residue for argininyl-tripeptide hydrolysis, forms hydrogen bonding interactions with the guanidinium group of arginine to stabilize the N-terminal arginine residue and position the tripeptide for hydrolysis within the AP active site^28,31^. In addition, Arg563 and Lys565 are key residues for tripeptidase function as they cooperate with each other for strong alignment of the substrate at the binding site^32^. The crystal structure of [E296Q]LTA_4_H in complex with Arg-Ser-Arg (PDB ID: 3B7S) shows that the N-terminal arginine points toward Gln136^31^. The superposition of LTA_4_H:4MDM and [E296Q]LTA_4_H shows that the methoxy group of 4MDM likely hinders binding of Arg-X-X by physically clashing with N-terminal arginine atoms (Fig. S4), which agrees with our enzyme kinetics data that 4MDM inhibits N-terminal arginine substrate by a specific inhibition mechanism. The overlays of these X-ray crystal structures with the LTA_4_H:4MDM crystal structure demonstrates that 4MDM modulates peptidase activity through its influence on the hydrophobic interactions and rotational freedom of Q136. Also, the structural data suggest that nonpolar ligands are likely necessary for enhancing the peptidase activity due to more favorable binding entropies in the active site. Hence hydrophobic interactions and steric considerations are the primary factors influencing what modified kinetics may be possible. Hydrolysis of peptides with an N-terminus containing a smaller hydrophobic side chains are more likely activated by 4MDM, whereas larger or more polar side chains would likely be inhibited by 4MDM.

In conclusion, our studies demonstrate biochemical mechanisms of the LTA_4_H AP activity in details that were not previously attempted. Since the studies by Numao and, now, our studies are raising a possibility of more complex biological activities exerted by the LTA_4_H protein and its substrates^19^, a simple assessment of IC_50_ or AC_50_ to screen therapeutic molecules seems inadequate. Our data strongly suggest LTA_4_H AP activity, as modulated by 4MDM, is biologically relevant for anti-inflammatory responses. This response, however, appears independent of its action on PGP. These observations merit attention, because most previous methods seem incomplete and premature to determine even the simple therapeutic effects based on biochemical enzymatic activities. Development of any compounds targeting LTA_4_H will require complete enzymatic characterization as we have done in our recently published work^25^. Moreover, a single therapeutic agent such as 4MDM may experience substrate-dependent mechanisms, leading to highly divergent interactions (such as inhibition or activation) with LTA_4_H. This is clearly an underappreciated aspect of LTA_4_H biology. The potentially opposite therapeutic effects a single compound can generate in vivo complicates the search for therapies targeting LTA_4_H. More careful assessment and characterization of LTA_4_H enzymatic activities are much desired to further inform strategies for developing therapeutics for this important target. We recommend reexamining the mechanistic studies on inflammatory responses with regards to the complex activities of LTA_4_H and to base the discovery of new therapeutic agents on the distinct modification mechanisms of the enzyme.

## Methods

### Protein expression and purification

Human recombinant LTA_4_H with an N-terminal (His)_6_- and Xpress-tag was expressed in *E. coli* BL21(DE3) cells. Cells were cultured in LB medium containing 100 μg/mL ampicillin at 37 °C. Protein expression was induced with 1.0 mM isopropyl β-D-thiogalactopyranoside at OD_600_ = 0.6, and cells were grown for 20 h at 22 °C. Following harvesting, cells were resuspended in Histrap buffer A containing 10 mM imidazole, 200 mM NaCl, 10 mM Tris–HCl pH 8.0 and lysozyme. Cell lysates were clarified by centrifugation (14,000× *g* for 30 min) at 4 °C, then loaded onto a Histrap HP column (GE Healthcare). LTA_4_H was eluted with a linear gradient using Histrap buffer B containing 300 mM imidazole, 200 mM NaCl, and 20 mM Tris–HCl pH 8.0. LTA_4_H post Histrap was loaded onto a Mono Q column (GE Healthcare), washed with 10 mM Tris–HCl pH 8.0 buffer and eluted with a linear gradient of 0–0.5 M KCl containing 10 mM Tris–HCl pH 8.0 buffer. After elution, purified LTA_4_H was desalted using a desalting spin column (7 K Zeba spin column, Thermo Scientific) in 25 mM Tris pH 7.8 buffer, concentrated, then evaluated by SDS-PAGE to confirm purity.

### Crystallization, data collection and structure determination

For LTA_4_H:4MDM complex, LTA_4_H (27 mg/ml, MW = 69.3 kDa) was co-crystallized with 4MDM. Crystals were obtained from drops containing 60–75 mM magnesium formate dihydrate and 19–23% PEG3350 at 22 °C. For LTA_4_H:4MDM:OPB-Pro complex, LTA_4_H (15 mg/ml) was co-crystallized with N-(4-oxo-4-pyrrolidinyl-butanoyl)-proline (OPB-Pro, Aurora Fine Chemical, LLC) and 4MDM. Crystals were obtained from drops containing 60–90 mM magnesium formate dihydrate and 20–25% PEG3350 at 22 °C. Both crystal diffraction data were collected at 100 K in-house at the WRAIR X-ray Diffraction Facility using a Bruker Microstar rotating anode X-ray generator with a Pt 135 CCD detector. For data collection, crystals were cryoprotected in mother liquor with the addition of 25% ethylene glycol and frozen in a N_2_ gas stream. The data was reduced with the Proteum software from Bruker, and the structure was determined by molecular replacement using the program Phaser within the PHENIX suite. The LTA_4_H structure (PDB ID: 4MS6) was used as a search model for phasing following the removal of water and ligands. 4MDM and Zn^2+^ molecules were built within Fo-Fc density using coot with refinement in PHENIX.

### AC_50_/IC_50_ of 4MDM with Ala-pNA, Arg-pNA and Pro-pNA

For AC_50_/IC_50_ determination, 150 μL reactions contained 10 μg/ml LTA_4_H, 4MDM and 1xPBS pH 7.2. The concentration of 4MDM was varied from 0 to 8000 μM. After a 10-min incubation period at room temperature, 50 μL of 4.0 mM L-alanine-*p-*nitroanilide (Ala-*p*NA, Chem-Impex International Inc.), L-arginine-*p-*nitroanilide (Arg-*p*NA, Alfa Aesar), or L-proline-*p-*nitroanilide (Pro-*p*NA, Chem-Impex International Inc.) was added to the reaction for each experiment. The LTA_4_H AP enzyme reaction was continuously monitored at A_405_ for 15 min with 10 s intervals at 30 °C immediately following the addition of 50 μL of substrate solution. Replicates (*n* = 3) were run for each 4MDM combination.

### Kinetic assay with Ala-pNA, Arg-pNA, Pro-pNA and PGP in the presence of 4MDM

Assays were performed in 200 µL volumes containing LTA_4_H, 4MDM, and substrate in 1xPBS buffer pH 7.2 using a 96-well plate (Corning Costar). Ala-*p*NA, Arg-*p*NA, Pro-*p*NA solutions were prepared at various concentrations (0.5–8.0 mM) in the above buffer. 4MDM in 5% DMSO was added to each well at various concentrations (0–2.0 mM) and then 10 µg/ml LTA_4_H was added to the well with incubation at 30 °C for 10 min. Enzyme activity was measured by continuously monitoring the increase in absorbance at 405 nm for 30 min with 10 s intervals at 30 °C immediately following the addition of the substrate. Six replicates were run for each 4MDM and substrate combination and all readings were measured using a Bio-Tek Powerwave. PGP assays were performed at 30 °C in 1xPBS buffer pH 7.2 using a 96-well plate (Corning Costar). Proline-glycine-proline (PGP, Biomatik) was prepared at various concentrations (100–1000 μM) in the above buffer. 4-MDM dissolved in 5% DMSO were added to each well with various concentrations (0–0.32 μM) and then LTA4H (31.25 ng/ml) was added to 200 μL well for 30 min incubation at 30 °C. The enzymatic reactions were stopped at different time points, then the enzyme activity was measured using the fluorescamine derivatization method (Tecan Spark 10 M Spectrophotometer). The standard curve for Gly-Pro was determined, in advance, and used for enzyme activity data analysis.

### Murine model of acute lung inflammation induced by LPS

All methods were carried out in accordance to ARRIVE (Animal Research: Reporting of In Vivo Experiments) guidelines and relevant regulations. Ethical approval was granted from the University of Virginia Institutional Review Board (IRB) #3527. Pathogen-free 8–10 week old female C57BL/6 mice were purchased from the Jackson Laboratory. The Institutional Animal Care and Use Committee at the University of Virginia approved all experiments. All compounds are > 95% pure by HPLC analysis. Mice were anesthetized by IP ketamine and xylazine mix. All mice received intranasal (IN) lipopolysaccharide (LPS) (from *Escherichia coli O55:B5*, Sigma-Aldrich) diluted in 50 μL of sterile saline on Day 0 (20 μg/mouse), while one half of the mice received IN 4MDM in CDX-PBS (vehicle) and the other half received IN vehicle alone. Mice were then treated daily with IN 4MDM or vehicle. Mice were treated on Days 0 (baseline before exposure to LPS) then once daily intranasally on Days 2–4 (Fig. 1A). Whole lung bronchoalveolar lavage (BAL) was performed with our previously published method^6,33^. Cell pellets of the BAL fluid were stained with antibodies specific to CD45 labeled with PerCP (BD Bioscience), CD11b labeled with APCcy7 (BD Bioscience), and Ly6G labeled with PE (BD Bioscience). Flow cytometry was performed using previously published gating method^6,33^. Representative pictures of the H&E with peri-bronchial leukocytes infiltration were taken. Levels of LTB_4_ in BAL fluid were measured with a commercial ELISA kit (R&D systems). LTA_4_H AP activity in BAL fluid was measured by using a previously published method^6^.

### BALF sample cleanup for PGP quantification using GC/MS

BALF samples were first filtered to remove salts and unwanted biological components present within the sample matrix. This removal was accomplished through the use of Waters C-18 Plus Short Cartridge Sep-Paks, with 360 mg of sorbent (Waters Coporation). Briefly, cartridges were conditioned by preforming three washes with acetonitrile (FisherScientific) followed by three washes with a 0.1% (v/v) trifluoroacetic acid (TFA) (Chem-Impex Internation) in MilliQ water solution, using a 1 mL Air-Tite All-Plastic Norm-Ject syringe (Air-Tite Products Co Inc) to dispense each solvent. BALF samples were then acidified with 1 μL of TFA, after which 300 μLs were drawn up and pushed through the cartridge at a rate of 1–3 drops per second. An additional two washes with the 0.1% (v/v) TFA in MilliQ water solution was then performed to remove any components in the sample matrix not compatible with the LC–MS analysis. Finally, bound PGP was eluted from the cartridge using a 20% (v/v) acetonitrile in MilliQ water solution. Eluate was dried in a SpeedVac, and resuspend in 300 μLs of a 0.1% (v/v) formic acid (FA) (Sigma Aldrich) in MilliQ water solution.

### GC/MS instrumental analysis

PGP quantification was carried out on a TSQ Quantum Ultra Triple Quadrupole mass spectrometer (ThermoFisher Scientific) connected to an Accela HPLC pump (ThermoFisher Scientific). Samples were injected onto a 1 mm × 150 mm Hypersil Gold 3 μm particle C-18 reversed phase column (ThermoFisher Scientific) using an Accela autosampler (ThermoFisher Scientific). Mobile phase A consisted of 0.1% FA in MilliQ water. Mobile phase B consisted of 0.1% FA in methanol. An isocratic gradient of 90% A and 10% B at a flow rate of 50 μLs min^−1^ was employed for the first 6 min of the run. Then, following the primary elution of PGP at 3.5-min mark, the gradient shifted to 100% B and held there for an additional minute before returning to initial conditions for the duration of the run, allowing for column regeneration. The mass spectrometer was run in the selected reaction monitoring (SRM) mode to detect PGP present in BALF samples. Transitions pairs used for SRM analysis have been provided in Table S3. Stock solutions of PGP were prepared from lyophilized PGP (Bachem, Bubendorf, Switzerland) in phosphate buffered saline (PBS) pH 7.4 (ThermoFisher Scientific) at six concentrations, ranging from 156 to 5000 pg mL^−1^. Standards were prepared fresh and extracted the same day as BALF samples to ensure consistent processing.

### Statistics

Prism v9.2.0 (Graphpad) was used for all statistical analyses. Murine study results were analyzed by two-way ANOVA with Bonferroni corrected subgroup analyses. P valued less than 0.05 was considered significant. Enzyme kinetic samples were prepared in six replicates (*n* = *6*), and percent consumptions for each well was calculated and selected for the initial velocity using the *p*NA standard curve line of best fit. The coefficient of variation is within 5%.

## Supplementary Information

Supplementary Information.

## Data Availability

The data that support the findings of this study are available in the Supplementary Information file, and from the corresponding authors upon request. Coordinates and structure factors for all structures have been deposited to the Protein Data Banks, with the accession numbers of 7KZE and 7LLQ.

## References

[1] Samuelsson B (1982). The leukotrienes: an introduction. Adv. Prostaglandin Thromboxane Leukot. Res..

[2] Nijkamp FP, Sitsen JMA (1982). Leukotrienes, allergy and inflammation. Pharm. Weekbl. Sci..

[3] Corless J, Paracha M (2002). The use of leukotriene modifying drugs in asthma and other respiratory diseases. Curr. Drug Target Inflamm. Allergy.

[4] Lee TH (1984). Effects of exogenous arachidonic, eicosapentaenoic, and docosahexaenoic acids on the generation of 5-lipoxygenase pathway products by ionophore-activated human neutrophils. J. Clin. Invest..

[5] Snelgrove RJ (2010). A critical role for LTA4H in limiting chronic pulmonary neutrophilic inflammation. Science.

[6] Paige M (2014). Role of leukotriene A_4_ hydrolase aminopeptidase in the pathogenesis of emphysema. J. Immunol..

[7] Snelgrove R, Kheradmand F (2014). Leukotriene A_4_ hydrolase: the janus enzyme shows its ugly side in smokers. Am. J. Respir. Crit. Care Med..

[8] Wells JM (2014). An aberrant leukotriene A_4_ hydrolase–proline-glycine-proline pathway in the pathogenesis of chronic obstructive pulmonary disease. Am. J. Respir. Crit. Care Med..

[9] Orning L, Fitzpatrick FA (1992). Albumins activate peptide hydrolysis by the bifunctional enzyme LTA4 hydrolase/aminopeptidase. Biochemistry.

[10] Nissen JB, Iversen L, Kragballe K (2006). Characterization of the aminopeptidase activity of epidermal leukotriene A4 hydrolase against the opioid dynorphin fragment 1–7. Br. J. Dermatol..

[11] Griffin KJ, Fitzpatrick FA, Gierse J, Krivi G (1992). Opioid peptides are substrates for the bifunctional enzyme LTA4 hydrolase/aminopeptidase. Prostaglandins.

[12] Michael Y, Paige M, Khatami M (2012). Leukotriene A4 hydrolase: an evolving therapeutic target. Inflammatory Diseases – Immunopathology, Clinical and Pharmacological Bases.

[13] Thunnissen MMGM, Nordlund P, Haeggström JZ (2001). Crystal structure of human leukotriene A4 hydrolase, a bifunctional enzyme in inflammation. Nat. Struct. Biol..

[14] Low CM (2017). The development of novel LTA4H modulators to selectively target LTB4 generation. Sci. Rep..

[15] Davies DR (2009). Discovery of leukotriene A4 hydrolase inhibitors using metabolomics biased fragment crystallography^†^. J. Med. Chem..

[16] Snelgrove RJ (2011). Leukotriene A4 hydrolase: an anti-inflammatory role for a proinflammatory enzyme. Thorax.

[17] O’Reilly P (2009). N-α-PGP and PGP, potential biomarkers and therapeutic targets for COPD. Respir. Res..

[18] Abdul Roda M (2015). The matrikine PGP as a potential biomarker in COPD. Am. J. Physiol. Lung Cell. Mol. Physiol..

[19] Numao S (2017). Feasibility and physiological relevance of designing highly potent aminopeptidase-sparing leukotriene A4 hydrolase inhibitors. Sci. Rep..

[20] De Oliveira EO (2011). Effect of the leukotriene A4 hydrolase aminopeptidase augmentor 4-methoxydiphenylmethane in a pre-clinical model of pulmonary emphysema. Bioorg. Med. Chem. Lett..

[21] Braber S (2011). Cigarette smoke-induced lung emphysema in mice is associated with prolyl endopeptidase, an enzyme involved in collagen breakdown. Am. J. Physiol. Lung Cell. Mol. Physiol..

[22] Orning L, Gierse JK, Fitzpatrick FA (1994). The bifunctional enzyme leukotriene-A4 hydrolase is an arginine aminopeptidase of high efficiency and specificity. J. Biol. Chem..

[23] Baici A (2015). Kinetics of Enzyme-Modifier Interactions.

[24] Lin Y (2001). Substrate inhibition kinetics for cytochrome P450-catalyzed reactions. Drug Metab. Dispos. Biol. Fate Chem..

[25] Lee KH (2019). Effect of modifier structure on the activation of leukotriene A_4_ hydrolase aminopeptidase activity. J. Med. Chem..

[26] Haeggström JZ (2004). Leukotriene A4 hydrolase/aminopeptidase, the gatekeeper of chemotactic leukotriene B4 biosynthesis. J. Biol. Chem..

[27] Stsiapanava A (2014). Binding of Pro-Gly-Pro at the active site of leukotriene A4 hydrolase/aminopeptidase and development of an epoxide hydrolase selective inhibitor. Proc. Natl. Acad. Sci..

[28] Rudberg PC, Tholander F, Thunnissen MMGM, Samuelsson B, Haeggstrom JZ (2002). Leukotriene A4 hydrolase: Selective abrogation of leukotriene B4 formation by mutation of aspartic acid 375. Proc. Natl. Acad. Sci..

[29] Lin M (2008). Matrix metalloproteinase-8 facilitates neutrophil migration through the corneal stromal matrix by collagen degradation and production of the chemotactic peptide Pro-Gly-Pro. Am. J. Pathol..

[30] O’Reilly PJ (2009). Neutrophils contain prolyl endopeptidase and generate the chemotactic peptide, PGP, from collagen. J. Neuroimmunol..

[31] Tholander F (2008). Structure-based dissection of the active site chemistry of leukotriene A4 hydrolase: implications for M1 aminopeptidases and inhibitor design. Chem. Biol..

[32] Thangapandian S, John S, Lazar P, Choi S, Lee KW (2012). Structural origins for the loss of catalytic activities of bifunctional human LTA4H revealed through molecular dynamics simulations. PLoS ONE.

[33] Shim YM (2010). Role of LTB4 in the pathogenesis of elastase-induced murine pulmonary emphysema. Am. J. Physiol. Lung Cell. Mol. Physiol..

[34] Englert L (2010). Displacement of disordered water molecules from hydrophobic pocket creates enthalpic signature: Binding of phosphonamidate to the S1’-pocket of thermolysin. Biochim. Biophys. Acta (BBA) Gen. Subj..

[35] Tholander F, Roques B-P, Fournié-Zaluski M-C, Thunnissen MMGM, Haeggström JZ (2010). Crystal structure of leukotriene A _4_ hydrolase in complex with kelatorphan, implications for design of zinc metallopeptidase inhibitors. FEBS Lett..

[36] Armbruster DA, Pry T (2008). Limit of blank, limit of detection and limit of quantitation. Clin. Biochem. Rev..

[37] Pfister RR (2000). Synthetic complementary peptides inhibit a neutrophil chemoattractant found in the alkali-injured cornea. Cornea.

[38] Weathington NM (2006). A novel peptide CXCR ligand derived from extracellular matrix degradation during airway inflammation. Nat. Med..

[39] Brock TG (2005). Nuclear localization of leukotriene A4 hydrolase in type II alveolar epithelial cells in normal and fibrotic lung. Am. J. Physiol. Lung Cell. Mol. Physiol..

[40] Shrivastava A, Gupta V (2011). Methods for the determination of limit of detection and limit of quantitation of the analytical methods. Chron. Young Sci..

[41] Pluskal T, Castillo S, Villar-Briones A, Orešič M (2010). MZmine 2: Modular framework for processing, visualizing, and analyzing mass spectrometry-based molecular profile data. BMC Bioinform..

[42] Patel DF, Snelgrove RJ (2018). The multifaceted roles of the matrikine Pro-Gly-Pro in pulmonary health and disease. Eur. Respir. Rev..

[43] Wittmann SK (2016). Thermodynamic properties of leukotriene A 4 hydrolase inhibitors. Bioorg. Med. Chem..

[44] Stsiapanava A, Samuelsson B, Haeggström JZ (2017). Capturing LTA _4_ hydrolase in action: insights to the chemistry and dynamics of chemotactic LTB _4_ synthesis. Proc. Natl. Acad. Sci. USA.

[45] Markert C (2021). Discovery of LYS006, a potent and highly selective inhibitor of leukotriene A_4_ hydrolase. J. Med. Chem..

[46] Jiang X (2010). Modulating the substrate specificity of LTA4H aminopeptidase by using chemical compounds and small-molecule-guided mutagenesis. Chem. Eur. J. Chem. Bio..

[47] Jiang X (2008). Activation and inhibition of leukotriene A4 hydrolase aminopeptidase activity by diphenyl ether and derivatives. Bioorg. Med. Chem. Lett..

[48] Haeggström JZ (2000). Structure, function, and regulation of leukotriene A_4_ hydrolase. Am. J. Respir. Crit. Care Med..

[49] Fitzpatrick FA, Lepley R, Orning L, Duffin K (1994). Suicide inactivation of leukotriene A_4_ hydrolase/aminopeptidase. Ann. NY Acad. Sci..

[50] Byzia A, Haeggström JZ, Salvesen GS, Drag M (2014). A remarkable activity of human leukotriene A4 hydrolase (LTA4H) toward unnatural amino acids. Amino Acids.

[51] Penning TD (1995). Kelatorphan and related analogs: potent and selective inhibitors of leukotriene A4 hydrolase. Bioorg. Med. Chem. Lett..

[52] Gaggar A (2008). A novel proteolytic cascade generates an extracellular matrix-derived chemoattractant in chronic neutrophilic inflammation. J. Immonol..

