# Supplementary material for: Substrate-dependent modulation of the leukotriene A4 hydrolase aminopeptidase activity and effect in a murine model of acute lung inflammation
Source: Sci Rep. 2022 Jun 8;12:9443. doi: 10.1038/s41598-022-13238-6 (PMC9177663; doi:10.1038/s41598-022-13238-6)
Supplement: Supplementary file 1 — Supplementary Information. [file 41598_2022_13238_MOESM1_ESM.docx]

**SUPPORTING INFORMATION:**

Table S1. Selected reaction monitoring pairs for PGP transitions corresponding to y1 and y2 ions, collision energy (CE), and collision gas for fragmentation.

| Parent | Product | Collision Energy (CE) | Collision Gas |
| --- | --- | --- | --- |
| 270.000 | 173.010 | 15 | He - 1.5 mTorr |
| 270.000 | 116.010 | 15 | He - 1.5 mTorr |

Table S2. PGP levels in BALF of LPS murine model from our data and other published data. Our data indicated that [PGP] is independent of LTA_4_H AP activity over time. ^*^Detection limit is 0.11 ng/mL and limit of quantitation is 0.62 ng/mL.

| Our data (ng/mL)^*^  C57BL/6 mice | | Blalock’s data^38^ (ng/mL)  BALB/C mice | | Numao’s data^20^ (ng/mL)  BALB/C mice | |
| --- | --- | --- | --- | --- | --- |
| 24 hr | 0.22 | 24 hr | 2.40 | 24 hr | 7.63 |
| 48 hr | 0.16 | 48 hr | 0.20 | 48 hr | 15.26 |
| 120 hr | 0.30 |  |  |  |  |


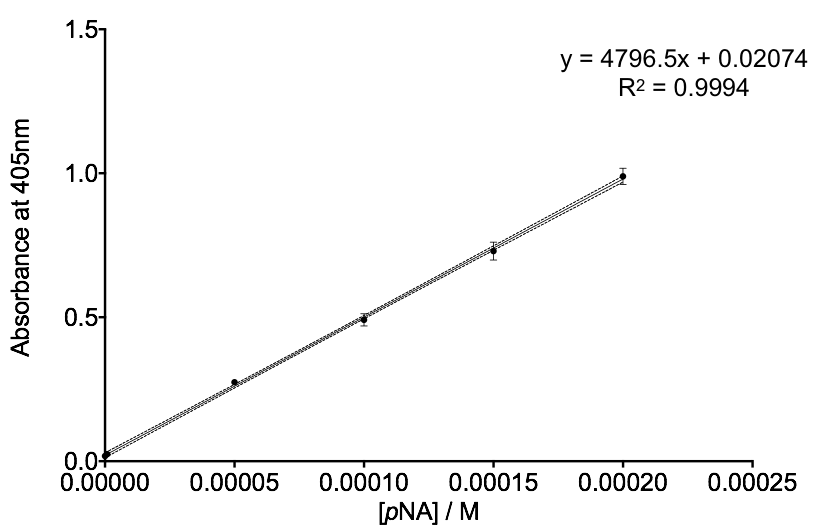


Figure S1. Standard curve of *p*-NA. The standard curve for para-nitroaniline quantification was generated by measurement of the chromophore prepared from commercially available para-nitroaniline. The reading of each diluted sample was obtained at λ=405 nm. The limitations of the plate reader were statistically analyzed (Table S3). The line of best fit is shown to convert the reading from the plate reader into the molar concentration of *p*NA. The bars represent ± standard deviation from nine replicates (*n=9*).

Table S3. Analysis of the limitations of the plate reader. The critical level (LC) of the plate reader is 0.018 at λ=405 nm with a blank reading, which provides the range for the signal corresponding to a *p*NA concentration of zero. The limit of detection (LD) is the response of the sample that is above the limit of blank (LC) with a confidence of 95%. The LD value for *p*NA is 0.023, which is the lowest level of detection for *p*NA. The limit of quantification (LQ) is 0.031, which is the functionally sensitive response for the lowest *p*NA concentration that is above the LD.^30^

| The critical level, L_C_ | 0.018 |
| --- | --- |
| The limit of detection, L_D_ | 0.023 |
| The limit of quantification, L_Q_ | 0.031 |

Figure S2. Molecular mechanism of Ala-*p*NA, Arg-*p*NA, and Pro-*p*NA hydrolysis. LTA_4_H AP activity hydrolyzes amino acid-*p*NA to an N-terminal amino acid and *p*NA.

Table S4. AC_50_/IC_50_ of 4MDM with Ala-*p*NA, Arg-*p*NA, and Pro-*p*NA. Data are represented as mean ± standard deviation of six replicates (*n=6*).

| Substrate | AC_50_ (μM) | IC_50_ (μM) |
| --- | --- | --- |
| Ala-*p*NA | 4.83 ± 0.02 | - |
| Arg-*p*NA | - | 328.10 ± 1.27 |
| Pro-*p*NA | 462.81 ± 0.43 | - |


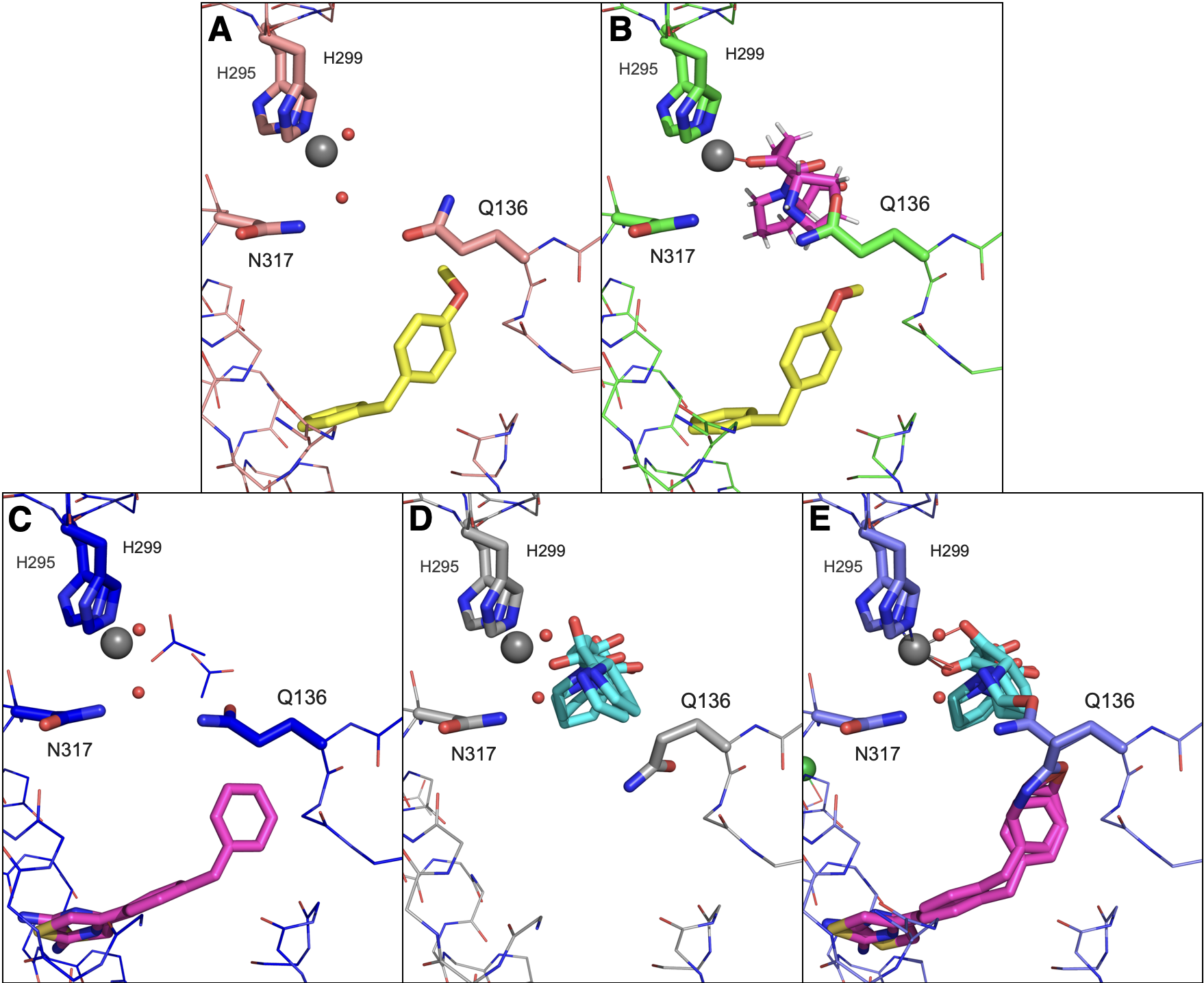


Figure S3. Enlarged view of LTA_4_H binding pocket in complex with (A) 4MDM, (B) 4MDM and OPB-Pro, (C) ARM1 (PDB ID: 4L2L), (D) OPB-Pro (PDB ID: 4MS6), and (E) ARM1 and OPB-Pro (PDB ID: 4MKT). There are two non-catalytic water molecules presented between Q136 and N317 in all structures except LTA_4_H:4MDM:OPB-Pro.


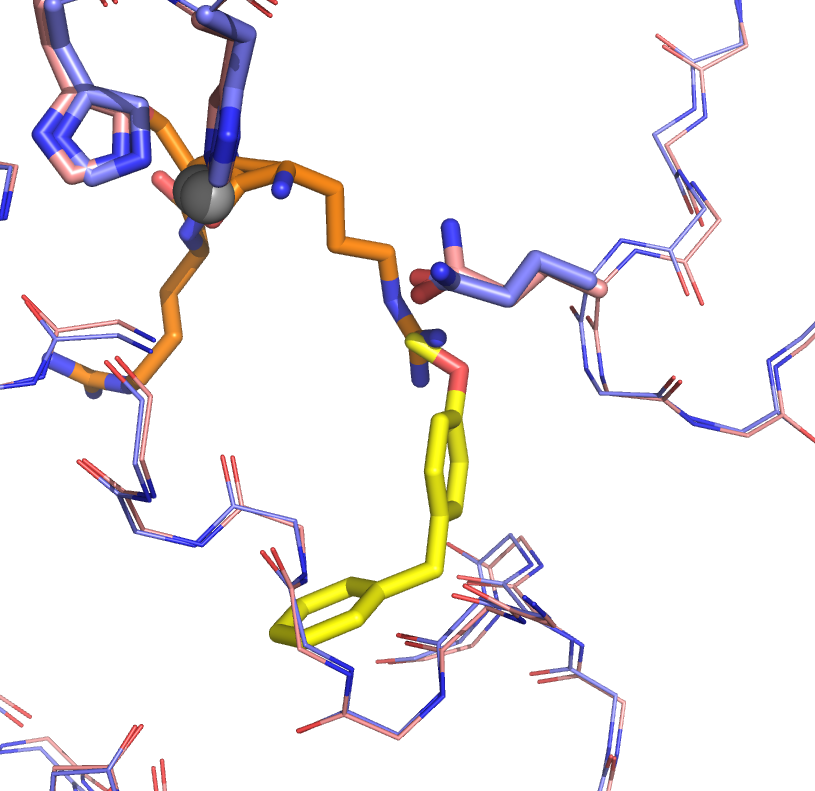


Figure S4. Superposition of LTA_4_H:4MDM and [E296Q]LTA_4_H (PDB ID: 3B7S).^24^ The methoxy group of 4MDM (yellow) is predicted to hinder Arg-Ser-Arg (orange) binding via steric clashes with the sidechain of the N-terminal arginine.
